# American Dental Society of Europe

**Published:** 1887-12

**Authors:** George Cunningham

**Affiliations:** Lecturer on Operative Dental Surgery at the National Dental College; Lecturer on Dental Surgery approved by the Special Board of Medicine, University of Cambridge.


ARTICLE II.

### AMERICAN DENTAL SOCIETY OF EUROPE.

### ADDRESS BY THE PRESIDENT OF THE SECTION ON DENTAL EDUCATION:

GEORGE CUNNINGHAM,

[Lecturer on Operative Dental Surgery at the National Dental College; Lecturer on Dental Surgery, approved by the Special Board of Medicine, University of Cambridge.]

GENTLEMEN:—Before proceeding to discuss the special subject with which this section is charged, permit me to offer a few words of explanation as to the position I now occupy. I need scarcely say how much I value the compliment of being asked to preside over so important a section as that on dental education, a compliment which I did not accept without a great deal of hesitation and some considerable amount of pressure on the part of some of the pillars of this Society, and that mainly for two reasons. The first was, because of my temperament, which makes me rather incline to the *role* of a free lance in the field of discussion than to that of President, whose qualities should always be associated with calmness, impartiality and the weight of great experience. My second reason was perhaps the more important of the two, namely, the possibility of my being unable to be present at the meeting. I am sure, however, that in the opinion of this Society my excuse is of the best; if possible I hope at or about the time of this meeting to be in Washington, taking part in the International Medical Congress.

In order to make the communications and the discussions on the subject of dental education as practical as possible, let me first of all direct your attention to the international aspect of the question. No doubt the old adage, "Circumstances alter cases," in which we are, perhaps, apt to seek refuge when a point is made against our cherished institutions or our pet theories and ideas, will come into play. Now, while we admit the great force of modifying circumstances affecting our different countries, it cannot be gainsaid that there is an ideal education common to the whole dental profession, irrespective of country, irrespective of existing or non-existing institutions, and irrespective of our personal predilections.

The aim and object of our proceedings, however, must be no vain effort in search of a fugitive ideal, but an earnest endeavour to embody our highest aspirations in a practical utilitarian form to show what in our opinion constitutes the minimum course of dental education which, in the best interests of the public, we should demand from our dental students.

As life itself is characterised by the three several stages of childhood, youth and manhood, so I think we can observe three corresponding periods in the educational development of the dental practitioner. We must consider, firstly, the standard of Preliminary Education, which we may rightly require of everyone pretending to enter a liberal profession such as ours; secondly, that Professional Education, both medical and dental, which is necessary to qualification; and thirdly, that most fruitful but too often greatly neglected course of all—the period of Post-graduate study.

These three stages of our subject could only be efficiently treated by a longer discussion than circumstances permit; and as I have no doubt a practical unanimity will prevail amongst us as to the first and possibly as to the third stages here indicated, I shall ask you, as far as possible, to concentrate the discussion upon the second stage, which we will again subdivide for the purpose of facilitating debate.

With regard to the first division of the subject, it stands to reason that the dental profession, as such, will never occupy its proper position so long as it fails to exact from its students the same amount of ordinary school education as is demanded of those entering other liberal professions.

Both in England and in Germany, I think, it will be admitted that the certificate of the preliminary examination in arts in the one country, and the certificate of efficiency for the prima in the other, is all that may be required, and has the additional advantage of being identical with that demanded of the medical student.

I trust I am only expressing the opinion of all present when I say that the dental profession will only be satisfied by the same standard of preliminary education being demanded of the dental student as is required of the medical student; in this respect, at least, we must be content to take no subsidiary place, and if in certain countries, notably

America, no such evidence of preliminary education is demanded of the medical student, the dental profession of that country will only be honouring itself by insisting upon evidence of such an initial preparation from its students, and so setting a good example to their medical brethren. I can well remember being greatly struck by this initial but essential difference between the dental student in England and in America, so much so that when called upon to respond for England at a social gathering of the American Dental Association in Philadelphia, in the year 1876, I made that very subject the principal theme of my speech. In justice to the great common and free school institutions of that country, every dental and medical faculty should demand an examination in arts equivalent to those already mentioned. A preliminary examination in arts would do very much to enhance the appreciation of American diplomas abroad.

The provisions of the English law, so far as regards preliminary education, possess one great advantage over the similar provisions in Germany. Besides having one specially recognised examination, they publish a list of certificates or diplomas attainable in different parts of the world which are recognised as equivalents, and what is still more important, such a qualifying examination is always open to a man, no matter what his age or what his antecedents, thereby enabling him to make up for the possible deficiencies of his earlier education. Owing to lack of this early training in many otherwise well qualified men, it might be well if some temporary provision could be made for men of tried experience and mature years who are desirous of proceeding to a diploma. The examination, which is fairly easy to a lad fresh from his school studies, becomes an almost insurmountable barrier to the man of some age, even if he has had a good scholastic training in his youth. The cramming up of such studies for examinational purposes must be of very equivocal value. In founding new regulations, some regard should be had for these individuals, not merely in justice to them, but to prevent the loss to the profession of many valuable recruits.

Secondly: the professional education of the dental practitioner may be considered under two heads, that which is general or medical and that which is special or dental.

The consideration of the first of these divisions is, I think, one on which this Society should express no undecided opinion. The main question resolves itself into this: shall it be identical with that of the medical practitioner, or shall it be limited to a part only of the full medical curriculum? To many this question may seem scarcely worth discussion, from the general consensus of dental opinion in favour of the latter view; and so far as England indeed is concerned, after a long and vigorous contest on the part of the opposition, the question may be considered to be finally settled. It cannot be denied, however, that a number of the most earnest workers in dental research, and many of the best dental practitioners on the Continent, strongly maintain the necessity of the dental surgeon being fully qualified medically. With all sympathy for this noble ideal, I have a very strong conviction that the firm adherence to the medical curriculum as a *sine qua non* is a tremendous obstacle to the establishment and advancement of a reasonable and an efficient dental curriculum. This is notably so in the case of such countries as France, Austria and Hungary, where the barren discussion still goes on, while the special dental curriculum of England and of Germany is bearing a rich harvest.

The aphoristic statement that "the greatest good is that of the greatest number" should alone suffice to convince those who hold this lofty but narrow view on dental education. In these days, the urgent and almost universal demand for the services of the dental practitioner has called into existence an immense army of more or less unqualified men, especially where no special dental diploma has been instituted. In the interests of the public, it is our duty to agitate for such an institution, and, when obtained, to maintain its honour and integrity with all our might. Considering the very exceptional and special nature of the necessary dental training for the efficient practice of what is, and must ever be, an important but yet minor branch of general surgery, it is manifestly absurd to contend that this specialty should only be practised by those who have expended about half as much again of the time and cost of study as is required of the major practitioner, in whose hands we are content to leave the very issues of life and death itself.

Though I have used the term minor as applied to our specialty, it must not be inferred that minor education or minor examination will satisfy our aspirations. The original memorial addressed to the Royal College of Surgeons of England, for the institution of the dental diploma in 1860, strikes the right note and is as true to-day as it ever was. "The memorialists do not suggest an education and examination inferior to that required of the medical practitioner, but proposes a certain advantage in kind only, not an advantage in degree—an education and examination especially adapted to the requirements of the dental surgeon as distinguished from that for the general surgeon." The two greatest authorities who have ever written on dental education strongly support the contention we are now making. The first is the present distinguished President of the Harvard University, who, in an admirable address, estimated the general medical subjects at three-fifths and the special at two-fifths of the whole education, rightly required after the aspiration to a dental diploma. The other, and not less distinguished authority, is that of Sir John Tomes. A careful perusal of his address on "The Study of Dental Surgery, and the means thereto," delivered at the International Congress of 1881, should be sufficient to convince all those earnest supporters of the cause of dental education who still maintain the necessity of a full medical curriculum for the dental practitioner that in so doing they are wrong, and that by modifying their views in consonance with his, and by promulgating them, they will be rendering a service alike to themselves, to their profession and to their country.

If we decide, then, that the general or medical part of the curriculum required of the dental practitioner shall not be identical with that of the medical practitioner, we must carefully consider what subjects should be common to both curricula. So far as the consideration of this part of the subject is concerned, we cannot do better than consider the minimum requirements of the General Medical Council, England:

TABLE OF MEDICAL SUBJECTS FOR THE DENTAL DIPLOMA.

| | | | |
|---|---|---|---|
| Anatony - - | One Course of Six Months.* | | |
| " - - | Dissections—Nine Months. | | |
| Physiology - | One Course of Lectures—Six Months. | | |
| Chemistry - - | " | " | " |
| Practical Chemistry | " | " | Three Months. |
| Surgery - - | " | " | Six Months. |
| Medicine - - | " | " | " |
| Materia Medica | " | " | Three Months. |
| Practical Surgery and Clinical Lectures—Two Winter sessions of Six Months each. | | | |

Anatomy, physiology and chemistry constitute the essential foundation of any medical education. Therefore, we fail to see why the general primary medical education and examination of the dental student should not be identical with that required of the medical student. It must be admitted that this further similarity as to the primary examination would do much to prove that the dental "curriculum" only differs by kind and not in degree from the medical, and consequently would do much to greatly im-

* The Royal College of Surgeons of England require a second course of Lectures on Anatomy, or a course of 20 Lectures on Head and Neck.

prove the position of our profession. Under existing conditions in England, such a step ought to be readily effected, as the class-attendance already required so nearly coincides with that of the medical student. In anatomy, for instance, the dental as compared with the medical student is relieved of only a part of one course of lectures and of three months' dissection. Again, the dental student is excused attendance on the thirty meetings of the practical physiology class No such exemption should be allowed, as the remarkable advances made in physiology require a longer period of study than that at present allotted in the dental curriculum. The attendance on the lectures on chemistry and the three months' practical course is identical for both the dental and the medical student in England; but unfortunately the former is not examined on the subject, the natural result being that he too frequently neglects to make a proper study of the subject.

Examination in chemistry should form an essential part of the curriculum, unless indeed it becomes, as in some of our university curricula, a part, with biology and physics, of the preliminary scientific or first M. B. examination. The studies and examinations at Harvard University, so far as regards anatomy, physiology and general chemistry, are absolutely identical with that required from the medical student. I cannot help thinking that physiology does not receive the attention it merits at the examination board in England.

Those who are interested in ascertaining the place which physiology should rightly acquire, whether as a discipline or as practical useful knowledge, ought to read the able and eloquent address of Professor Michael Foster, delivered in Cambridge in 1880 before the British Medical Association.

One course of six months' lectures on surgery, on medicine, and three months on materia medica, also an attendance at a recognized general hospital, with clinical instruction of not less than one year, seem very reasonable provisions to demand from the dental student. The lectures on forensic medicine, midwifery, pathology, practical pharmacy and vaccination required in the medical curriculum are not demanded from dental students, but are replaced by special dental studies.

The only amendment which we could propose would be the exclusion of pathology from this list of exempted subjects. Thanks mainly to the teachings of Cohnheim and other pathologists of the German school, the study of general pathology is now regarded rather from the view of disturbances and derangements in function, and also of perverted nutrition, than from that of purely morbid anatomy and histology. It must be evident, therefore, that a complete and practical study of the elements of general pathology, with its allied and included subject of bacteriology, is essential to a due and proper comprehension of the special pathology of the parts with which the dental practitioner has to deal. A course on this subject would certainly be more useful than, and might advantageously replace, the three months' course on general materia medica.

We must now consider what Sir John Tomes rightly terms the all-important special subjects comprised in a dental curriculum. This part of the subject divides itself into two main brancles, viz., mechanical and operative dentistry, the latter term being used in its very widest sense.

So far as mechanical dentistry is concerned, the requirements of the English diploma are: first a certificate of attendance during a term of three years in the laboratory of a reputable dental practitioner; and, secondly, the attendance on two courses of lectures, on mechanical dentistry and the one course on dental metallurgy. In order that these lectures should have their full weight and importance with the dental student, they should be attended during his term of pupilage, and not, as is so frequently the case in England, after this period, when he has laid aside the sculptor and the file, of which he is probably somewhat tired, for the excavator and the plugger, with which he is more enamoured.

As Sir John Tomes so wisely and beautifully expresses it, "It is one thing to know the scientific principles of an art, but it is quite another to carry them into effect. This requires an amount of skill of hand which can be attained only by long and careful practice under competent teachers. The fingers must become unconsciously obedient to the will; they must follow it automatically as the fingers of the skilled pianofortist execute the mental reading of the work he is playing, or as the hand of the sculptor produces the form the mind has conceived. Short of this unbidden obedience of hand, the performer would be but an amateur, and his professional life one long apology—a life of words in the stead of work."

From the possibility of such a certificate being more or less fraudulent, from the fact that the instructor may be incompetent or neglectful, but mainly from the cause that in many countries the profession is now a closed one, it becomes a serious question whether or not the teaching in this subject should not be conducted in a school under the open method, as is already the case in operative dentistry. Though we might not think it advisable to abolish the older system, which has much to recommend it, the establishment of an optional course in an open school might excite a tendency to greater efficiency on the part of private instructors.

The seven years' apprenticeship in the good old-fashioned English "workshop," with its advantages as a mechanical training, is gone forever. It would seem easy, however, to resuscitate something, if not all, of the skillfulness of the dental mechanic of the past by allowing a part of the period of pupilage to be spent in a course of instruction in practical mechanics in some school of technology. By the adoption of some such pain, the student in the laboratory would acquire a training not merely mechanical in name but in fact.

The consideration of that part of our subject which relates to operative dentistry need not detain us long. In the English schools, the lectures are confined to two courses on both dental-anatomy and physiology, and on dental surgery and pathology. With regard to dental anatomy, I can confidently say it would be difficult to find a better and more complete course than that which exists in either of the two London Dental Schools, embracing as it does a very thorough and complete study of comparative odontology, and following closely the lines laid down in Tomes' "Manual of Dental Anatomy." It is difficult to assume that the study of the student in this subject can be complete until some provision is made for his having a previously acquired knowledge of animal morphology, which already forms a part of an English university medical curriculum.

The syllabus, too, of the lectures on dental surgery and pathology is complete, thorough and exhaustive. The question, however, may well be raised, whether the dental profession should still be content with mere didactic teaching on those subjects. I think there can be no doubt that a very considerable, if not the major, part of the advance in medical science is greatly due to the enormously improved system of physiological teaching in which didactic teaching no longer suffices without a thorough and complete practical course of study in the shape of exercises in the physiological laboratory. If in this respect we can further identify the system of teaching those two fundamental departments of special knowledge with the practical system now adopted in the medical schools, we might safely expect to further raise the special dental scientific knowledge of our profession and thereby promote our greater efficiency as practitioners of the healing art.

Attendance is not required on any special series of lectures on operative dental surgery by any of the British licensing bodies. While admitting that no mere attendance on such a course will suffice to make the dental student an efficient operator, I certainly think that a good course on this subject would do much to improve the dental student. In such a conrse it should be the aim to show the application of the many various methods and appliances now in use, to the various normal and abnormal conditions with which he has been familiarised by his training in the other courses of lectures. At the National Dental College of London such a course has been instituted for nine years, and I think the success which has attended the teaching of the two able lecturers who have held the post, namely, Dr. Finley Thompson and Dr. St. George Elliott, should stimulate the larger London Dental School to found a similar course of lectures.

The treatment of irregularities of teeth forms so important and essential a part of the dental practitioner's practice, that we do not think it receives adequate attention when it forms the subject of only a few lectures in the course of dental surgery and pathology. The appointment of a specialist in this subject to deliver a full and complete series of lectures and demonstrations on this subject would do much to raise the attainments of the profession in the treatment of this difficult part of our specialty. It is with deep sense of the advantages which I derived from such extra courses of lectures at the Harvard Dental School, including also one on dental materia medica and therapeutics, that I am prompted to suggest their adoption in our English schools.

With regard to the attendance at a recognized dental hospital, the provision of a full two years' course, as required by the British licensing bodies, is adequate. The teaching of the demonstrators is efficient and thorough, and as an evidence of the remarkable advance which the London schools have made in this respect, I cannot do better than refer to the evidence of one of your own Presidents, Dr. St. George Elliott, who said that, as an examiner, he had seen as good work in operative dentistry by English dental students as by the students of any American school

Thanks mainly to the exertions of the Dental Examiners at the Royal College of Surgeons of England, especially of Mr. Thomas Arnold Rogers, a practical examination forms an essential and important part of the ordeal necessary to obtain the English qualification. This part of the examination is conducted at one or other of the dental hospitals by the Dental Members of the Examining Board, who are in no way connected with the teaching department of the schools. A liberal supply of patients is provided, the student is required to explain fully the proper methods for the treatment of the mouths requiring operations, and is usually required to perform a more or less complicated gold filling. He must also give and discuss his opinion on the appropriate treatment of several cases of irregularity and other dental lesions. The examination mostly lasts about three hours, and from a recent personal experience, I can vouch for the extreme fairness, completeness and thoroughness of that part of the examination. The Deans of the London Dental Schools have advocated the addition of a practical exbmination in mechanical dentistry, a suggestion which commands approval.

With regard to the other part of the examination at the Royal College of Surgeons, England, it is conducted by papers and *viva voce* questions on anatomy and physiology, surgery and pathology, dental anatomy and surgery, and dental mechanics. The only improvements that we could suggest in this examination would be its division into two parts—the first for the purely medical, the other for the dental subjects. In this way it would seem possible to make some provision for examination in some of the subjects taught but not examined upon, such as chemistry, medicine, and materia medica.

The only other certificates required by the British licensing bodies to which allusion has not been made is that, first, of being twenty-one years of age; and, second, of having been engaged four years in professional studies, after passing the Preliminary Examination.

I think, from the information I have given with regard to the English diploma, you will be convinced that, while we may be of an opinion that it is capable of improvement and further development, it is the highest, most extensive and complete dental curriculum which exists. I think you will also admit that the four years required is none too much in which to do justice to such a curriculum, and indeed I know that a very large number of the students are unable to overtake it within that period of time.

In countries where a special dental diploma is a new institution, and the requirements pressing; it may be contended, with no little show of reason, that such a course is too long, too onerous and too expensive. It may be well, however, to remember that the English curriculum is the same as originally instituted in the year 1858, with the exception of the preliminary examination, which was wisely only made obligatory on students beginning their studies after the passing of the Dentists' Act of 1878. Of course, with the enormous advance in teaching, very considerable improvement has taken place in the development and practical results obtainable by such a standard or dental education. We cannot but think, therefore, that the nearer the various schools approximate the English standard the better it will be for the dental profession and the general public in all countries.

This is one excellent feature in the English scheme to which I must allude, and that is the formation and publication of a complete register of dental practitioners by the General Medical Council of Great Britain, A similar system of registration should form an essential part of the scheme wherever a dental diploma is instituted.

With regard to the third and final period of dental education, the period of post-graduate study has not, it seems to me, yet met with adequate recognition at the hands of those who have written, many of them so well and so ably, on dental education. The development of this part of the subject I must leave to a more fitting opportunity, and will merely indicate one or two points which merit attention.

The following out of the relatively small amount of extra study which would enable the dental practitioner to acquire a full medical and surgical diploma may be strongly recommended to those who are ambitious to take high places in the profession, and who have the time and the money to spare, as both will be profitably invested. It is also a question whether a further extension of special dental teaching might not be instituted for the benefit of those who would prefer to still further develop a scientific study of their specialty. Such courses, being instituted under the guidance of the best workers in the profession, might take the line of investigation and original research.

At Edinburgh University, a course of post-graduate study has been arranged for the benefit of medical practitioners, and in connection with it, I believe that the Edinburgh Dental School purposes instituting similar courses of study for dental parctitioners already in practice.

Until the institution of some such course or courses as we have indicated, the young dental practitioner should early avail himself of the existing substitute by associating himself with dental as well as other scientific societies. He should not merely be content with the enjoyment of the social advantages to be derived from such institutions, but he should also make a serious study of the papers and discussions with a view of taking an early and intelligent part in the same. If in any way he can attach himself to a general or special hospital, whether in the capacity of dental surgeon or demonstrator, he will be doing much to promote his final and truly higher professional education.

From actual personal experience, I should also strongly recommend the young dental practitioner not to be too eager to rush into practice for himself, and provided the opportunity occur, he will do well to associate himself as assistant to some dental practitioner of repute and good practice. Here he will find an opportunity of eradicating some of the minor shortcomings which seem incidental to hospital practice, and if not too bumptious and full of his own conceit, he will thereby secure an experience which is not to be measured by the amount of his emoluments.

One often hears a good deal against the Irish diploma granted *sine curriculo.* Let me call your attention to one good and excellent provision which the Irish College of Surgeons exacts from its graduates: "I shall not attract business by advertising or any other unbecoming practice, and I agree that such diploma shall be cancelled on its being proved that I have done so." * Like many another evil thing, the advertising quack will be ever with us, but a united and well-directed effort should be made to restrain the graduates of all reputable dental schools from debasing their diploma, their school and their profession, by advertising and other disreputable and unprofessional means of attracting practice.

In conclusion, gentlemen, I apologize to you for the lengthy nature of this address, and I can only plead in extenuation the nature of the subject, which will no doubt meet with the exhaustive discussion its great importance merits.—*London Dental Record.*

---

ARTICLE III.

### SOFT GOLD FOIL.

GEO. H. WINKLER, M. D., D. D. S.

[Read before the Southern Dental Association at Old Point Comfort, Va., September 1st, 1887.]

In offering this essay on gold foil, I shall confine myself to a discussion of that form of foil which is rendered

* Something similar to this is agreed to by the Licentiates in Dental Surgery for the Edinburgh College and of the Faculty of Glasgow, but not by the College of Surgeons of England.

